# Memristive Motion‐Streak Neuron for Spatiotemporal Multiple Object Detection

**DOI:** 10.1002/adma.74793

**Published:** 2026-08-22

**Authors:** Hyungjun Park, Jin Hong Kim, Hyun Wook Kim, Néstor Ghenzi, Sung Keun Shim, Joon‐Kyu Han, Min Jung Chung, Dong Hoon Shin, Kyung Seok Woo, Cheol Seong Hwang

**Affiliations:** ^1^ Department of Materials Science and Engineering and Inter‐University Semiconductor Research Center Seoul National University Seoul Republic of Korea; ^2^ Graduate School of Semiconductor Materials and Devices Engineering Ulsan National Institute of Science and Technology (UNIST) Ulsan Republic of Korea; ^3^ Department of Materials Science and Engineering Ulsan National Institute of Science and Technology (UNIST) Ulsan Republic of Korea

**Keywords:** dynamic memristor, dynamic vision sensor, motion streak neuron, neuromorphic computing, optoelectronic memristor, spatiotemporal processing

## Abstract

Conventional artificial vision systems process dynamic scenes inefficiently by reconstructing motion from discrete frames, which requires post‐processing. In contrast, real‐world environments containing multiple moving objects demand sensor‐level discrimination. This work presents a memristive motion‐streak neuron that performs spatiotemporal encoding by integrating an Al/InGaZnO/Al optomemristor with an Ag/HfO_2_/Pt dynamic memristor, whose relaxation dynamics provide temporal memory. In this system, the presence time of moving objects is detected by decay of the output current, allowing motion direction and speed to be directly inferred from the relaxation behavior. The integrated memristor pixel array enables processing of continuous movements and achieves 96.2% classification accuracy for multiple objects. Also, integrating the motion‐streak neuron with the resistor–capacitor kernel further encodes temporal intervals between optical events, enabling recognition of complex movement patterns. This dynamic processing diminishes computational overhead and provides a hardware solution for next‐generation vision systems.

## Graphical abstract

This work proposes motion‐streak neuron hardware using amorphous In–Ga–Zn–O optoelectronic memristors and dynamic memristors for spatiotemporal multiple‐object detection. The motion streak neuron array processes optical and electrical stimuli, allowing speed‐dependent filament relaxation to encode motion trajectories. This capability advances neuromorphic vision hardware toward in‐sensor processing in dynamic visual scenarios.

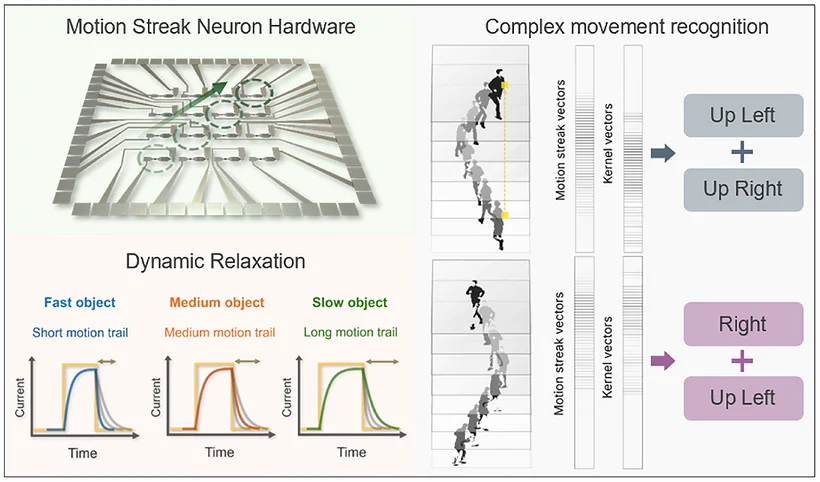

## 1 Introduction

Recent advancements in artificial intelligence have significantly improved machine vision performance by mimicking biological vision systems [1, 2, 3, 4, 5]. However, most machine vision systems are predominantly designed for static image perception through a frame‐by‐frame approach rather than continuous dynamic scenes [6, 7, 8], resulting in redundant data transfer and high latency, particularly when multiple objects move simultaneously at different speeds and in different directions. Therefore, dynamic vision systems capable of simultaneously analyzing spatiotemporal information are crucial for efficiently handling massive video data [9].

The biological visual system does not reconstruct dynamic scenes from discrete frames [10, 11], but instead integrates dynamic visual inputs as a continuous stream by overlaying image frames to form a single, integrated visual scene, thereby enabling perception of complex visual scenarios [12, 13, 14]. A key component of this spatiotemporal integration is a motion‐streak neuron that combines successive visual inputs into residual traces of moving objects, enabling the processing of continuous visual data and rapid motion perception. Inspired by this principle, neuromorphic memristive motion detection and perception systems require elements that simultaneously exhibit stimulus responsiveness and intrinsic dynamic memory, enabling the direct encoding of complex motion features within the device.

This work introduces a memristive motion‐streak neuron by integrating an Al/InGaZnO (IGZO)/Al optomemristor with an Ag/HfO_2_/Pt dynamic memristor, forming a unit that functions as an individual pixel and captures spatiotemporal data from multiple objects. The integrated memristor pixel (neuron) enables learning from video data using a single embedded frame, without processing each frame individually. A 4 × 4 motion‐streak neuron array was fabricated, and its operational principle was confirmed by illuminating the devices with laser pulses of controlled direction and speed (duration). Subsequently, the KTH dataset was simulated to demonstrate that the integrated system can accurately recognize the direction and speed of multiple moving objects, comparable to existing software solutions [15, 16]. Furthermore, integration with a resistor–capacitor (R–C) kernel allows the proposed neuron to detect complex motion of multiple objects, supporting scalable motion‐processing applications. This study presents a method that bridges the gap between artificial and biological vision systems.

## 2 Results and Discussion

### 2.1 Motion Streak Neuron with Dynamic Relaxation for Spatiotemporal Multiple Object Detection

Figure 1a illustrates how the motion‐streak neurons in the human cerebral cortex integrate temporal frames from multiple objects into a single visual frame, where multiple frames were combined to form residual traces to infer the speed and direction of moving objects [17]. Direction is determined by the object's position in the final embedded frame relative to its residual trace. Also, speed is characterized by the length of these traces; fast‐ and slower‐moving objects leave shorter and longer traces, respectively [18].

**FIGURE 1:**
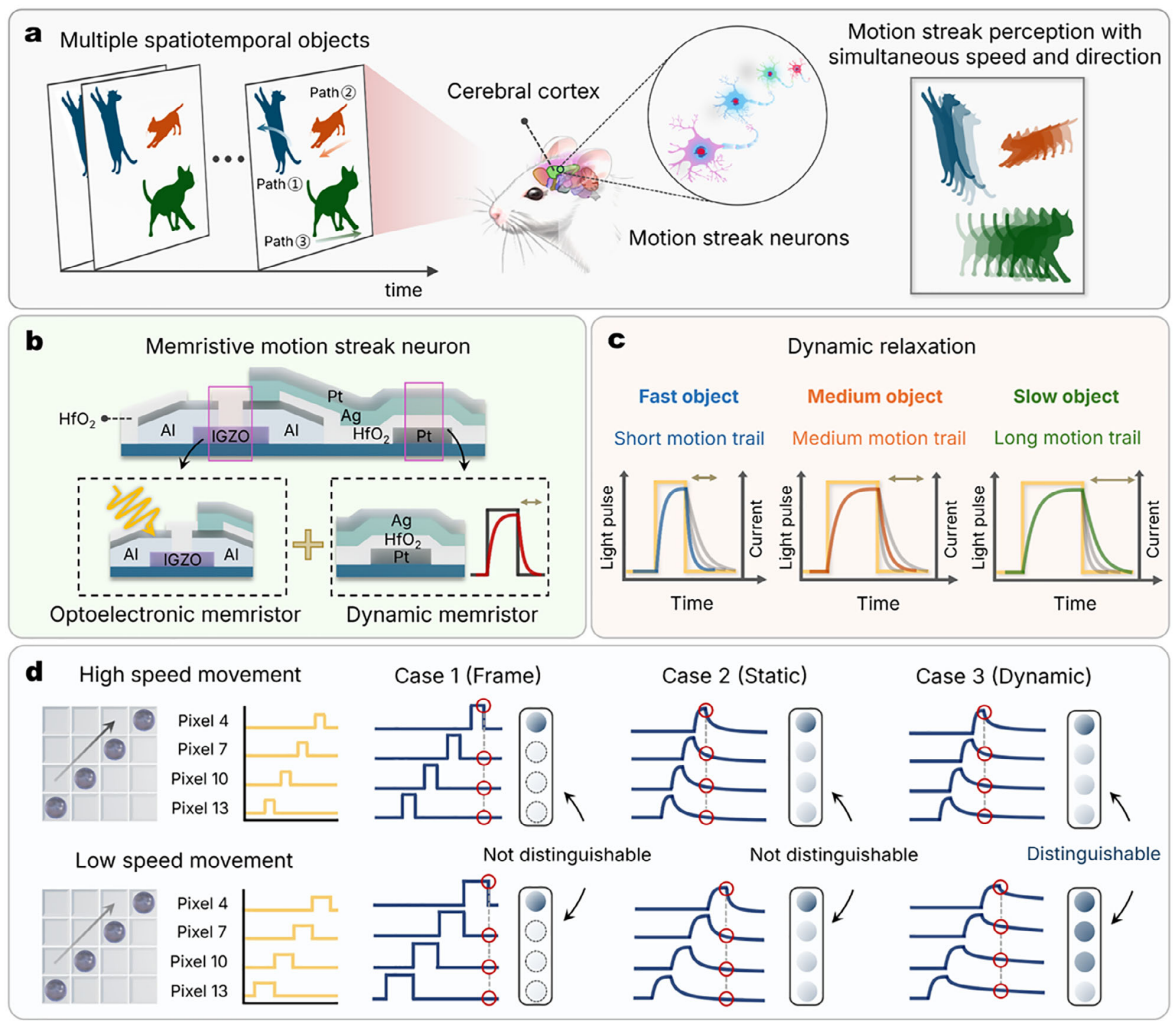
Illustration of a motion streak neuron for spatiotemporal multiple object detection. (a) Biological motion streak neurons in a mouse cerebral cortex. Motion‐streak neurons are featured in simultaneously recognizing the speed and direction of multiple objects. (b) A motion streak neuron device consisting of an Al/IGZO/Al optoelectronic memristor and an Ag/HfO_2_/Pt dynamic memristor. The optoelectronic memristor converts external optical stimuli into electrical currents, and the dynamic memristor exhibits relaxation characteristics that encode object velocity as motion streaks. (c) The relaxation time of the memristive motion streak neuron increases with the input pulse width. When the integrated device directly receives light, generated motion trails can capture the speed characteristics of multiple objects. (d) By encoding the object presence time into the optical pulse width, motion streaks can be clearly distinguished through dynamic relaxation. In contrast, frame‐based approaches without relaxation dynamics and static approaches lacking temporal correlation cannot distinguish velocity‐dependent features.

Figure 1b presents an implementation of a memristive motion‐streak neuron composed of Al/InGaZnO/Al optoelectronic memristors and Ag/HfO_2_/Pt dynamic memristors, emulating the biological motion‐streak neuron. Light pulses activate optoelectronic memristors, which primarily encode the spatial position of a moving object, while dynamic memristors are triggered by electrical pulses and capture the object's speed [19]. Figure 1c illustrates how distinct motion streak patterns emerge from the relaxation behavior of the dynamic memristor when light pulses are applied to the integrated device. In this method, the duration of a moving object's presence at a pixel is represented by the light pulse duration. A fast‐moving object corresponds to a short light pulse, resulting in rapid decay of the dynamic memristor current and the formation of a short motion trail. In contrast, a slow‐moving object is represented by a longer light pulse, which induces a prolonged relaxation process and generates extended motion streaks.

Figure 1d presents a 4 × 4 array of motion‐streak neurons with different operational principles. In a conventional frame‐based approach, each light pulse is considered as a single frame, and the frames are processed independently. As a result, dynamic motion is segmented into discrete segments, and thus, objects moving at different speeds cannot be reliably distinguished. In the static approach, motion trails are observed as a function of pulse length. However, the length of the motion trail (or decay time constant) does not vary with the object presence time (or light pulse length). Therefore, object speeds cannot be distinguished when objects move simultaneously. In contrast, the proposed dynamic approach modulates the intrinsic relaxation dynamics of the dynamic memristor. Distinct motion trails are generated based on the object's presence time at each pixel: longer decay time for longer presence (or pulse application) time. Through these properties, clear and simultaneous discrimination of object identity, motion speed, and direction is achieved without additional signal processing.

### 2.2 Electrical and Optical Properties of the Ag/HfO_2_/Pt Dynamic Memristor, Al/IGZO/Al Optomemristor and the Integrated Device

The Ag/HfO_2_/Pt dynamic memristor was investigated to emulate the temporal processing behavior of biological motion‐streak neurons [20]. Figure 2a shows 50 cycles of current‐voltage (I–V) characteristics of the dynamic memristor. After the forming process, the dynamic memristor exhibits abrupt volatile switching behavior below 1 V (Figure S1). When an electrical pulse was applied, the circuit exhibited a relaxation response due to the memristor's inherent parasitic capacitance, as shown in Figure 2b, which depicts the typical relaxation behavior under a constant‐voltage pulse. The Ag/HfO_2_/Pt device exhibits pulse‐dependent volatile relaxation through the formation and spontaneous dissolution of nanoscale Ag conductive filaments. The active Ag electrode supplies mobile Ag ions for filament formation, while the chemically inert Pt counter electrode minimizes unintended interfacial redox reactions and provides a well‐defined interface for Ag‐ion reduction and filament nucleation. These filament dynamics allow the relaxation time to vary with the pulse width and applied electrical conditions. Unlike abrupt transition‐dominated volatile switching, the Ag‐filament dynamics provide continuously tunable relaxation that reflects the preceding pulse history. The switching from a low‐conductance state to a high‐conductance state in the memristor is due to the formation of a silver conductive filament induced by the migration of silver cations under an electric field. When the pulse terminates, the device relaxes to a low‐conductance state (volatile off‐switching) via spontaneous rupture of the conducting filaments, driven by their surface energy [21, 22, 23, 24, 25].

**FIGURE 2:**
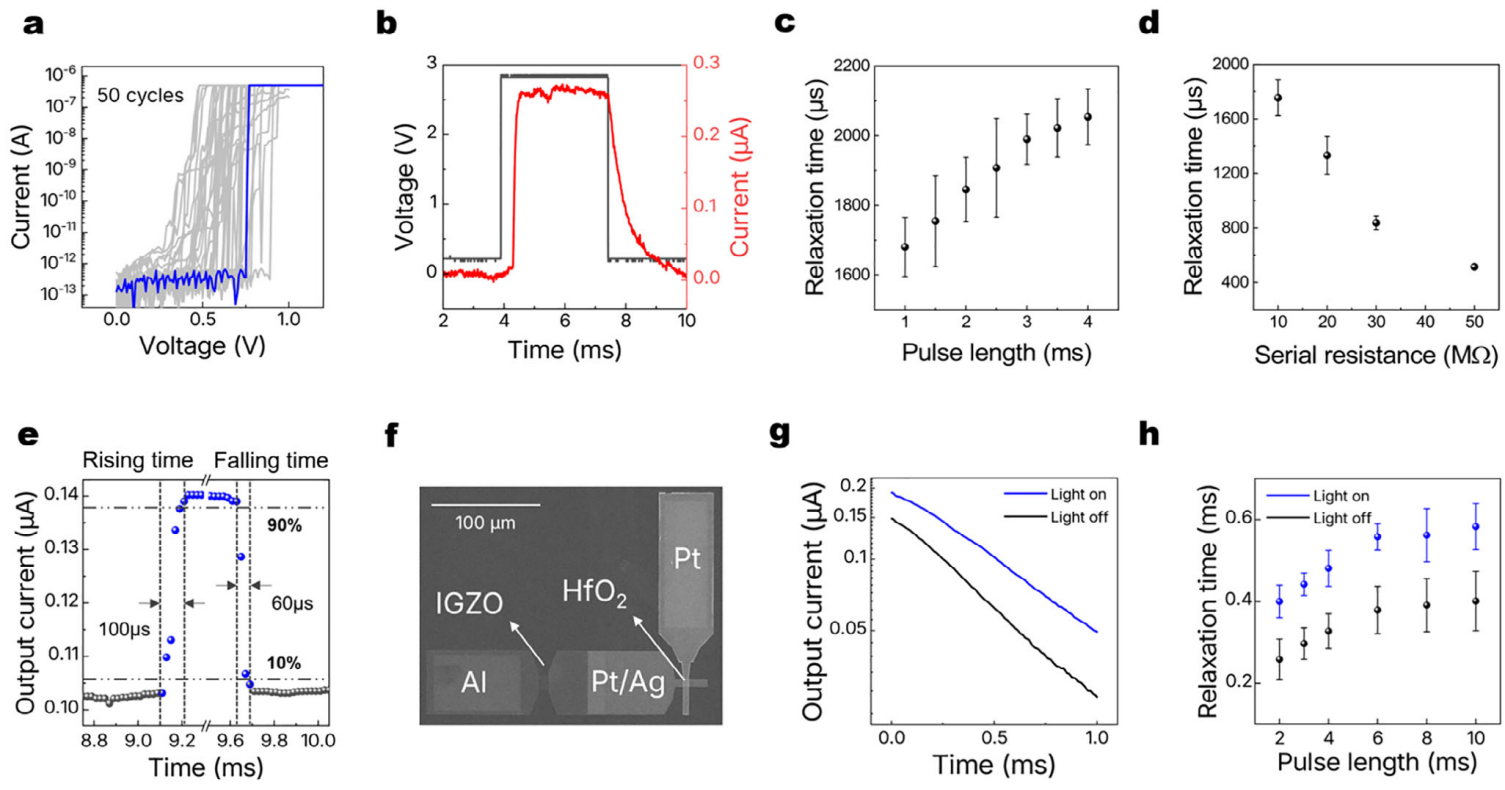
Electrical and optical properties of the Ag/HfO_2_/Pt dynamic memristor, Al/IGZO/Al optomemristor, and the integrated device. (a) DC current‐voltage (I‐V) characteristics of the dynamic memristor. (b) Threshold switching behavior of the dynamic memristor under an input pulse of 2.8 V over 2.5 ms. The output current (red) exhibits threshold‐switching behavior with relaxation dynamics. (c) Relaxation time at varying pulse widths under a fixed pulse voltage of 2.8 V and a load resistance of 10 MΩ, and (d) Relaxation behaviors at different series resistances under a fixed pulse condition (2.8 V and 2.5 ms). (e) Switching behavior of the optomemristor for incident light under a fixed voltage of 2.8 V. Rising and falling times are 100 and 60 µs, respectively. (f) Scanning electron microscopy image of the integrated device composed of the optomemristor and the dynamic memristor. (g) Relaxation behavior of the integrated motion streak neuron at different light conditions, with a fixed pulse condition (4 V and 8 ms), (h) relaxation time of the integrated motion streak neuron at different pulse widths and light conditions, with a fixed pulse amplitude of 4 V.

Figure 2c shows that the off‐switching relaxation time varied from 1.7 to 2.1 ms as the applied voltage pulse length increased from 1 to 4 ms, with a fixed pulse amplitude of 2.8 V and a serial resistor of 10 MΩ. Relaxation time is the time required for the device to return to zero after switching on. These varying relaxation times are attributed to differences in the diameter of the silver conductive filament. Figure 2d shows the results as the serial resistance increases from 10 to 50 MΩ under a fixed‐pulse condition (2.5 ms, 2.8 V). As the serial resistance increases, the voltage across the dynamic memristor decreases, decreasing the output current and shortening the relaxation time. Therefore, the Ag/HfO_2_/Pt dynamic memristor exhibits relaxation dynamics that depend on applied voltage pulse length and serial‐resistor conditions. Figures S2–S5 show the additional electrical characteristics of the dynamic memristor. Also, Figure S6 presents the extracted relaxation times over an extended pulse length range of 2 to 10 ms and pulse heights of 2.8 to 4.0 V. The monotonic and distinguishable relaxation time response confirms the feasibility of reliable speed encoding across a wider operating range.

The Al/IGZO/Al optomemristor complements the dynamic memristor through rapid, reversible, and approximately linear photoinduced conductance modulation. Its photoresponse remains substantially faster than the millisecond‐scale relaxation of the Ag/HfO_2_/Pt device, allowing the IGZO device to transduce optical inputs without limiting temporal encoding. Moreover, its light‐dependent resistance range effectively modulates the voltage distributed across the serially connected dynamic memristor. Figure 2e exhibits a rapid photoresponse of the Al/IGZO/Al optomemristor, with a rising time of 100 µs and a falling time of 60 µs under a light pulse with an intensity of 12.35 mW/cm^2^ and a pulse length of 0.5 ms. These sub‐hundred‐µs responses are superior to those of previously reported oxide‐based photodetectors [26, 27, 28]. The rapid photoresponse indicates that the proposed device can detect rapid visual data and convert it into electrical signals with minimal delay. Figures S7 and S8 present the band diagram and temperature dependence of the optomemristor under light‐on/off conditions. Figure S9 shows a linear relationship between the output current and light intensity, which is essential for accurately distinguishing between varying light intensities in practical operations.

Also, Figure S10 confirmed the reliable performance of the optomemristor over a 1.5‐s period of consecutive 2‐ms light pulses. The light response originates from a reversible reaction involving the ionization (*V_O_* → $V_{O}^{2+}$ + 2*e*) for light on and neutralization ($V_{O}^{2+}+2e$→*V_O_*) for light off of an oxygen vacancy (*V_O_*) [19]. The amorphous IGZO thin film exhibits favorable optical properties, which are attributed to numerous localized trap sites (neutral *V_O_*). Furthermore, Figure S11 represents stable light‐switching endurance over 400 consecutive cycles under a 2.8 V electrical bias with successive 2 ms light pulses. Note S1 shows more detailed discussions about the electrical conduction mechanism of the optomemristor with and without light.

Figure 2f shows that an Al/IGZO/Al optomemristor was serially connected to the Ag/HfO_2_/Pt dynamic memristor in place of the serial resistor, representing a single motion‐streak neuron (pixel). Figure S12 shows the schematic fabrication process flow of the integrated motion‐streak neuron. Figure 2g illustrates the relaxation behavior of the integrated motion‐streak neuron under light‐on/off conditions. At a fixed pulse condition (4 V, 8 ms), the relaxation time increases under illumination because the optomemristor has a lower resistance, thereby increasing the voltage across the dynamic memristor and resulting in a higher output current and longer relaxation time. Figures S13 and S14 show the I–V characteristics and endurance of the integrated device, demonstrating its electrical stability.

Figure 2h demonstrates the photoresponsive characteristics that affect both the output current and the relaxation behavior. The output current and relaxation time increased under the light‐on condition at all voltage pulse lengths, confirming the ability to process spatiotemporal information. The output current and relaxation time of 16 devices were measured to compare variation across the integrated devices (Figures S15–S17). Previous studies on optomemristors for motion detection were limited by the static photoresponse of the active layers [29, 30, 31, 32]. In contrast, this device can simultaneously process object motion.

### 2.3 Single Object Movement Classification with Integrated Motion‐Streak Neuron Array

Figure 3a illustrates the custom optical board setup used to demonstrate the operation of the memristive motion‐streak neuron experimentally. Figure S18 shows the hardware configuration used to detect a single object moving in different directions. The Methods section and Note S2 provide detailed descriptions of the optical measurement setup.

**FIGURE 3:**
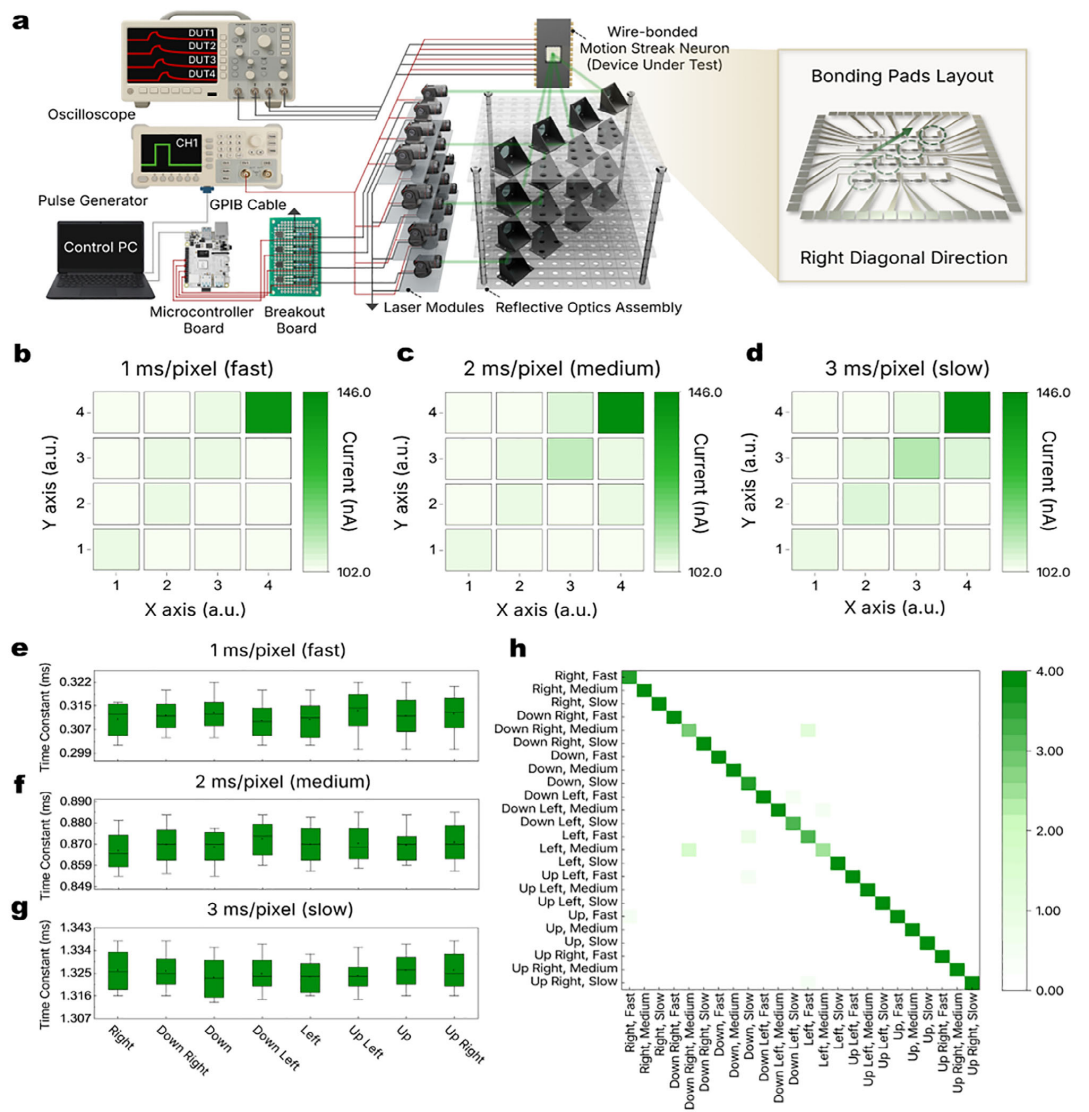
Hardware demonstration of motion streak neurons for single object movement classification. (a) Optical setup for precise timing control of the optical and electrical stimuli of the motion‐streak neuron hardware. The enlarged image on the right shows the layout of the 4 × 4 wire‐bonded motion‐streak neuron array, illustrating the application of light pulses along the right‐diagonal direction. Experimentally measured current of an object moving toward the right diagonal direction at different speeds: (b) fast (1 ms per pixel), (c) medium (2 ms per pixel), and (d) slow (3 ms per pixel). Relaxation time constants extracted from the measured current responses for objects moving in eight different directions, including vertical, horizontal, and diagonal trajectories: (e) fast (1 ms per pixel), (f) medium (2 ms per pixel), and (g) slow (3 ms per pixel). Each directional condition was measured over ten repeated trials. (h) Confusion matrix for single‐object movement classification.

To emulate the movement of a single object, optical pulse signals were applied with arbitrary directions and speeds, while a voltage of 4 V was applied simultaneously across all neuron devices. In this task, object speed is defined by the optical pulse length applied to each pixel, which is inversely proportional to the object velocity. Based on this relationship, three representative pulse lengths of 1, 2, and 3 ms per pixel were selected to denote the fast, medium, and slow speeds, respectively. Note S3 provides a quantitative relationship among object velocity, pulse length, and relaxation behavior.

For example, a slow‐moving object traveling to the right was implemented by applying four consecutive light pulses to the right at a rate of 3 ms per pixel, resulting in a total illumination duration of 12 ms. The direction and speed of the moving object were then determined by measuring the current responses of all motion streak neurons at the end of the 12 ms pulse sequence. Figure S19 shows the light‐pulse measurement system used to characterize the relaxation time of the motion‐streak neuron.

Figure 3b–d present experimentally measured current values obtained from the 4 × 4 motion‐streak neuron array for an object moving from the bottom left corner toward the top right corner along the diagonal direction at three different speeds. The color bar maps the current for each neuron, with darker colors indicating larger currents and lighter colors indicating smaller currents. Furthermore, the trade‐off between sensitivity and speed in current readout circuits can be mitigated through appropriate readout‐circuit design. For example, capacitive transimpedance amplifiers (CTIAs) and correlated double sampling (CDS) can enhance the detection of small current variations without significantly compromising the temporal resolution required for motion recognition. Video S1 presents a representative laser trajectory precisely controlled using the optical board setup. The spatial alignment between the laser spot and device pixels was quantified using the normalized distance between the laser centroid and the device active region. In addition, the timing resolution of the pulse generation system was analyzed by considering the major factors contributing to timing jitter. Figure S23 presents the spatial alignment ratios for individual device positions and the timing jitter analysis of the optical board setup. Each pixel reflects the neuron's relaxation response at its spatial location. For low (high) object speeds, the illuminated pixels retain stronger (weaker) residual currents when measured at the end of the light‐pulse sequence, enabling classification of both the direction and speed of moving objects. Figure S24 shows the results for motion trajectories beyond the right‐diagonal direction, confirming the motion‐selective capability of the proposed system. The relaxation dynamics of the dynamic memristor result in a temporal cost during signal readout, as a finite relaxation window is required to extract motion‐dependent temporal information. The readout integration time further affects the sensing speed, particularly when resolving small current variations. Therefore, the readout method should be optimized by balancing signal sensitivity and temporal resolution for real‐time motion recognition.

To assess the array's motion‐detection accuracy, training and inference were performed on simulated data derived from the experimental data. Figure 3e,f,g present the relaxation time constants extracted from the measured current responses for objects moving in eight directions at the fast, medium, and slow speeds, respectively. The decay time constant (τ) was determined by fitting the relaxation curve with $I\left(t\right)=I_{\mathrm{off}}+\left(I_{\mathrm{on}}-I_{\mathrm{off}}\right)*\exp\left(-t/\tau \right)$, where *I*_on_ refers to the current at the onset of the falling response after the laser pulse is removed, while *I*_off_ corresponds to the current after relaxation is complete. τ denotes the characteristic decay time required for the current to decrease to 1/*e* of its initial value after the stimulus is removed. The extracted relaxation time constants were consistent across different directions of the moving objects with the same speed and clearly separated for objects with different speeds. The classification was performed using the device current responses (motion‐streak patterns), while the fitted relaxation time constants were used to quantitatively analyze the speed‐ and direction‐dependent encoding mechanism. The discrimination margin can be further improved by modifying the relaxation dynamics of the dynamic memristor to achieve a wider tunable range of relaxation time constants. In addition, improved material uniformity and process control can reduce device variations, enabling more reliable motion discrimination under practical hardware operating conditions. Table S1 compares representative device characteristics for motion detection. The integrated device achieves fast rising/falling times, high endurance, and a high sampling frequency compared to other reported works. For each speed condition, relaxation time constants were obtained from 10 repeated trials for all motion directions. The measured relaxation values for each direction were flattened into a 16 × 1 column vector and fed to a neural network with 16 input neurons and 24 output neurons. Each output neuron label corresponds to a combination of motion direction and speed, such as an upright direction at slow speed. To more accurately classify single‐object movement, the neural network was trained by incorporating experimental current variations into the 16‐dimensional vectorized input patterns associated with each output neuron index [33, 34].

During the training phase, synaptic weights were randomly initialized and optimized through iterative updates to classify 16‐dimensional input vectors into 24 output neuron labels. Figure 3h shows clear diagonal patterns in the confusion matrix, with 24 labels corresponding to the combination of direction and speed. Figure S25 shows that test accuracy saturates at 96.7% after 80 epochs when selecting the highest‐output neuron. These results demonstrate that the arbitrary directions and speeds of the vectorized inputs can be clearly distinguished, even in the presence of noise induced by scattered light from moving objects. Video S2 shows the effect of laser crosstalk on adjacent neuron devices caused by scattered laser illumination.

### 2.4 Spatiotemporal Multiple Object Detection of the KTH Motion Dataset

The performance of the memristive motion‐streak neuron was further evaluated by simulating the KTH motion dataset, which features multiple objects moving in different directions and at different speeds. In this study, outdoor scenarios featuring human subjects running, jogging, and walking were selected from the KTH dataset [15, 16] to ensure a clear separation of speeds among the objects.

Figure 4a illustrates the construction of three arbitrary visual scenarios by combining two objects, eight motion directions, and three different speeds of 1, 2, and 3 ms per pixel. For each combined scenario, four motion events were sequentially applied, and the same process was repeated for the next scenario. Figure 4b shows that the current responses obtained after processing three motion scenarios on a 100 × 75 pixel array were vectorized into 7500 × 1 representations and subsequently classified using a neural network with 7500 input neurons and 48 output neurons. Here, each of the 48 output neuron labels corresponds to a unique combination of object identity, motion direction, and speed, such as object 1 moving in the up‐right direction at a fast speed. In addition, the experimentally measured coefficients of variation (CoV) for both cycle‐to‐cycle and device‐to‐device variations were used to sample device responses, enabling a realistic representation of device variability in the large‐scale array simulation. To validate the feasibility of multi‐pixel operation, device responses under repetitive 3 ms optical pulse trains were experimentally measured. Figure S20 shows stable, repeatable responses to repetitive stimulation, demonstrating the high reliability of the optomemristor and dynamic memristor for continuous video‐event processing tasks. Also, Figure S21 presents the current responses observed in neighboring pixels due to optical crosstalk. These characteristics were carefully incorporated to enable a more realistic evaluation of system performance. Furthermore, Figure S22 provides a quantitative assessment of optical crosstalk using the crosstalk ratio (CR), total crosstalk ratio (TCR), and signal‐to‐noise ratio (SNR).

**FIGURE 4:**
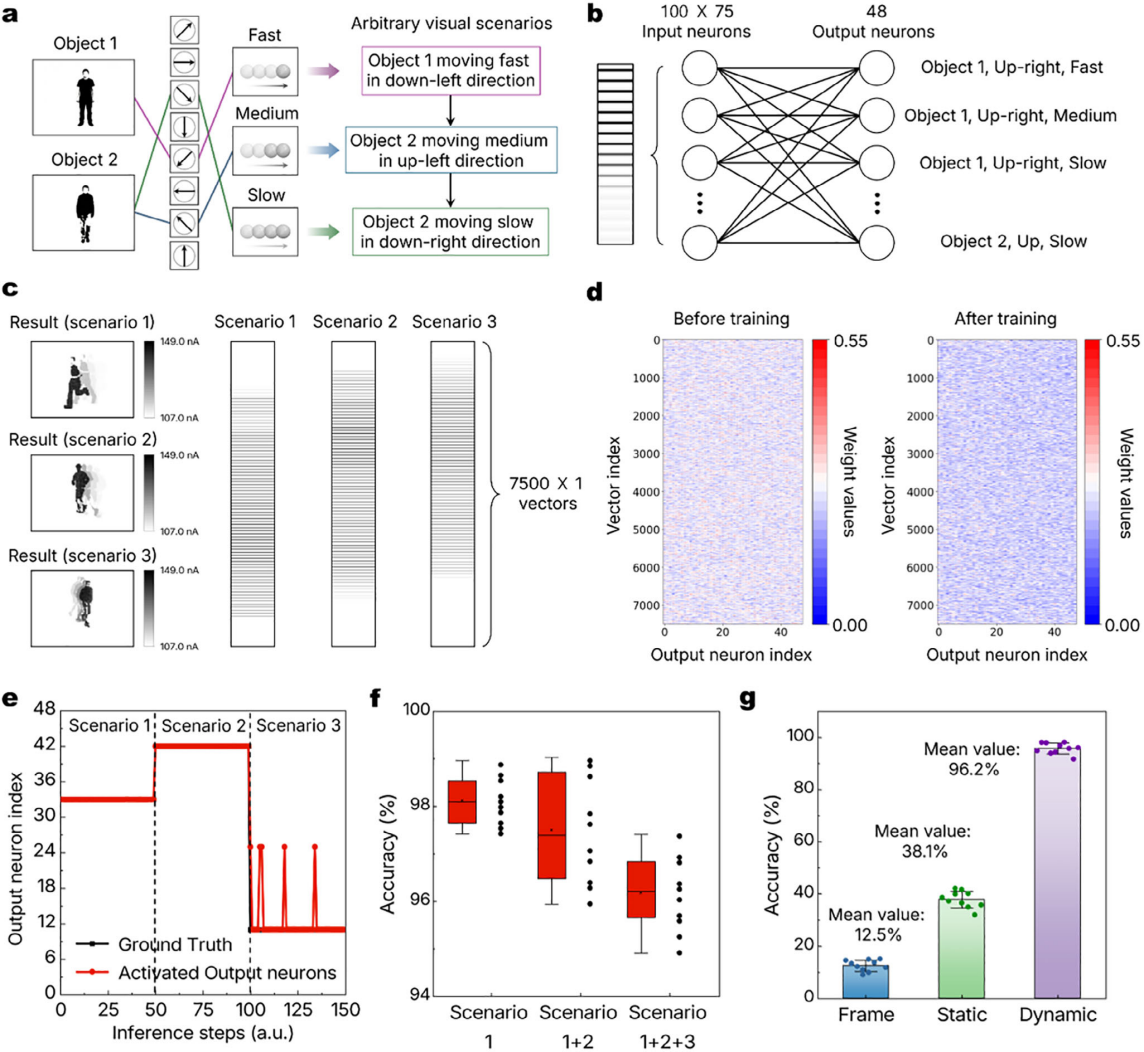
Spatiotemporal multiple object detection of the KTH motion dataset. (a) Arbitrary visual scenarios were constructed by combining two objects, eight motion directions, and three different speeds. Four sequential motion events were applied consecutively. (b) After each motion event, the measured current responses were processed by a neural network composed of 7500 input neurons and 48 output neurons for classification. (c) Processed results for three arbitrary visual scenarios. Distinct motion streaks were formed depending on each motion event, and the results were transformed to 7500 × 1 vectors. (d) Visualization of the neural network weight distributions before and after training. (e) Inference results of the output neuron indices for the three vectorized scenarios using the trained weights. (f) Inference results of the output neuron indices for accumulated scenarios containing one, two, and three visual scenarios. (g) Comparison of output neuron index classifications obtained using frame‐based, static, and dynamic approaches. Unlike the dynamic approach that distinct motion streaks enable clear index separation, the frame‐based and static approaches fail to resolve the output neuron indices.

Figure 4c presents the processing results for three arbitrary visual scenarios. Due to the dynamic relaxation behavior of the memristive neurons, distinct motion streaks emerge depending on both the direction and speed of each object. Slower (faster)‐moving objects in scenario 3 (1) produce motion trails with longer (shorter) streaks having higher (lower) intensity. For motions involving intermediate shape variations, such as posture changes during walking or jumping, multiple temporal samplings can be performed to capture additional motion states and encode the associated temporal information within the embedded frames.

Figure S26 demonstrates low optical crosstalk and sufficient pixel independence under simultaneous and temporally overlapping stimulation, enabling reliable separation of overlapping relaxation responses as the temporal delay increases. The 7500 × 1 vectorized representations of the current responses, obtained for each scenario are also shown in the right panel. Figure S27 presents the output vectors (7500 × 1) derived from motion‐streak neuron responses across three visual scenarios, shown for both frame‐based and static relaxation–based cases to enable direct comparison. Figure S28 compares the experimentally measured current response with the corresponding fitted result, confirming the validity of the device modeling process. Note S4 provides detailed descriptions of the constitutive equations and parameter extraction procedures. All scenarios inherently reflect device‐to‐device variations and relaxation dynamics, and each vector exhibits a unique pattern that enables classification into one of the 48 output neurons.

Figure 4d shows the distributions of synaptic weights before and after training. Initially, all weights were randomly initialized. However, after training, distinct weight patterns emerged for each output neuron, indicating that the network feasibly captured discriminative features associated with different motion streak patterns. Also, Figure S29 shows the learned weight distributions across output neuron indices after training for the frame‐based and static relaxation–based processing approaches.

Figure 4e shows the inferred output‐neuron indices for the three vectorized scenarios, accurately identifying the corresponding labels. Figure 4f compares inference accuracy across scenarios containing a single visual event (scenario 1), two combined events (scenarios 1 and 2), and all three visual scenarios. Consistent classification performance confirms that the proposed system maintains reliable inference even as the number of motion events increases. In contrast, conventional frame‐based sensors, which capture motion at a fixed frame rate, fail to capture continuous temporal dynamics within a single scene. Phototransistors with static relaxation characteristics were reported to partially address this limitation by reflecting dynamic changes in visual scenes [8, 35]. However, speed information could not be incorporated because relaxation behavior was not tunable. Figure S30 shows the classification outcomes for three arbitrary visual scenarios using frame‐based and static relaxation–based processing. Therefore, the capability to detect both direction and speed for individual actions demonstrates the suitability of the proposed system for classifying categories based on motion streaks associated with different actions. Figure S31 presents a confusion matrix for multiple moving‐object classification, demonstrating high accuracy across object identities, motion directions, and speeds.

Figure 4g compares the output neuron index classifications obtained using frame–based, static relaxation–based, and dynamic relaxation–based approaches. Frame–based sensing fails to capture temporal continuity, resulting in a low mean classification accuracy of only 12.5%. Static relaxation–based sensing partially reflects motion direction but cannot capture speed‐dependent variations, yielding a mean accuracy of 38.1%. In contrast, the dynamic relaxation–based approach enables clear separation of output neuron indices, achieving a substantially higher mean accuracy of 96.2%. The classification performance of the proposed neurons can be affected by device‐level variations, so classification accuracy was assessed as a function of the relaxation time coefficient of variation (CoV). The measured cycle‐to‐cycle and device‐to‐device CoVs were 0.09 and 0.07, respectively, yielding a total CoV of 0.12. As shown in Figure S32, test accuracy remains stable as CoV increases, indicating that variations in motion‐streak neurons do not significantly affect final classification performance.

### 2.5 Integrated Motion Streak Neuron and R‐C Kernel for Arbitrary Complex Movement Classification of Multiple Objects

To classify more complex movements involving multiple objects, it is also necessary to detect the temporal intervals between optical input sequences. Figure 5a illustrates multiple‐object scenarios with complex motion that extend beyond the simple scenarios used to evaluate arbitrary linear motion. For example, a movement in which an object first moves to the right and then moves to the left can be represented as a combination of right and left directions, while a movement that proceeds along the right diagonal after the left diagonal can be expressed as a combination of up‐left and up‐right directions. However, motion‐streak neurons cannot distinguish such complex sequential motions. In this case, the implications of the various output vectors could be further enhanced by using a physical kernel, such as the R–C kernel [36, 37], which nonlinearly transforms the input vectors into kernel vectors. These kernel vectors can then be combined with the input vectors to enhance classification accuracy.

**FIGURE 5:**
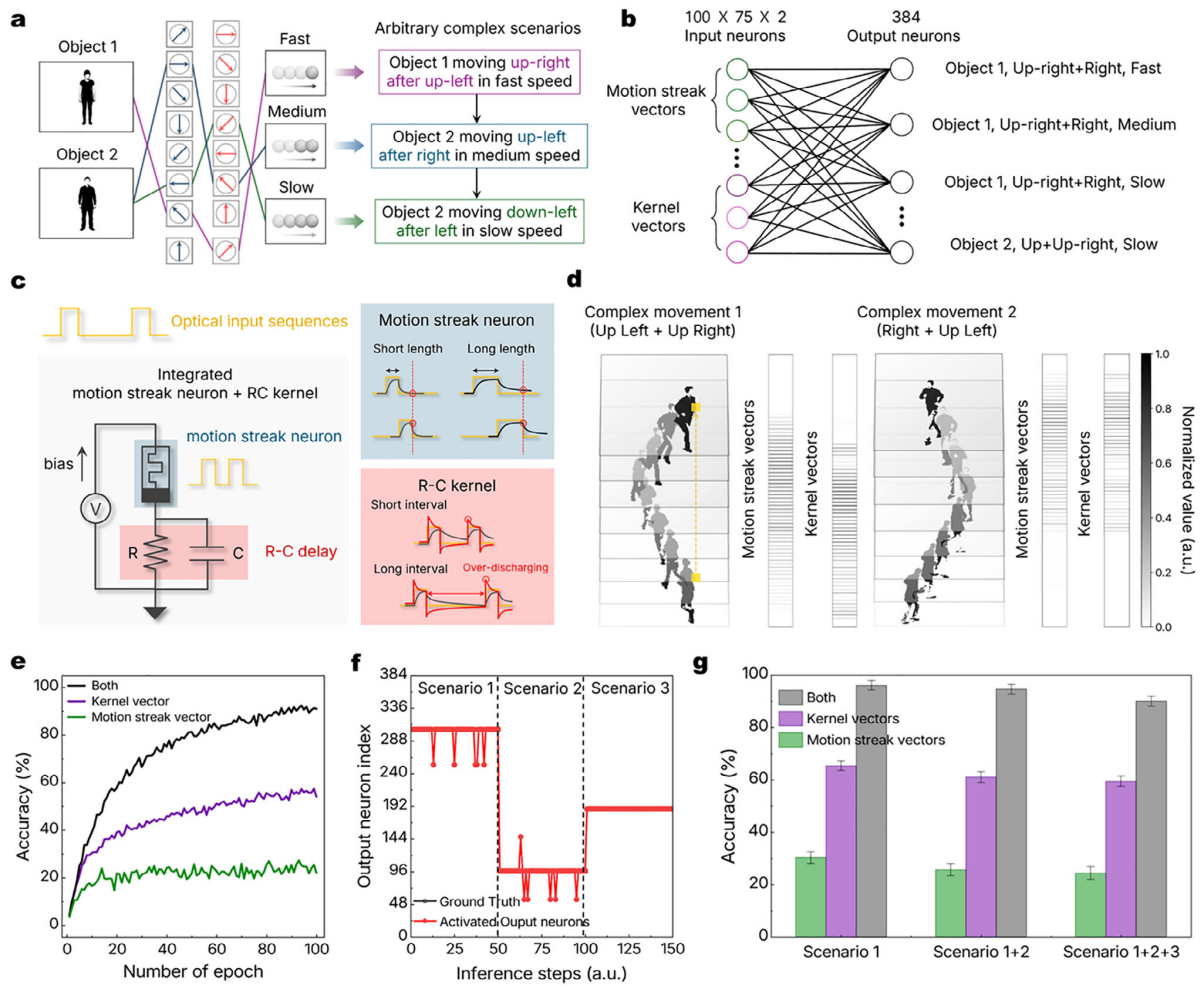
Integrated motion streak neuron and R‐C kernel for arbitrary complex movement classification of multiple objects. (a) Arbitrary complex scenarios were constructed by combining two objects, dual trajectories with eight motion directions, and three different speeds. (b) After complex movement scenarios, the motion streak vectors and R‐C kernel vectors from a 100 × 75 pixel region were vectorized into a 15000 × 1 vector and processed by a neural network with 15000 input neurons and 384 output neurons for classification. (c) Circuit diagram of the integrated motion streak neuron and R–C kernel. The motion‐streak neuron produces a motion trail proportional to the optical pulse width, and the R–C kernel encodes the temporal intervals in complex motion patterns. (d) Processed results for three arbitrary complex scenarios. Two vectors were formed independently, depending on the complex motion events. (e) Test accuracy as a function of epochs for three vectorized input cases. Compared with cases that use the motion streak vector or the kernel vector alone, combined vectors achieve significantly higher classification accuracy. (f) Inference results of the output neuron indices for the three vectorized inputs with motion streak vectors and R–C kernel vectors. (g) Comparison of test accuracy across three vectorized input cases under accumulated scenarios comprising one, two, and three complex visual events.

Figure 5b shows the structure of the readout layer designed to classify object identity, complex movement, and speed. In this configuration, optical input sequences applied to a 100 × 75‐pixel region determine the values of the motion‐streak neuron vectors and the R–C kernel vectors. These two vectors are flattened and concatenated to form a 100 × 75 × 2 input tensor. The 384 output neuron labels are defined by the combination of two object identities, eight motion directions 1 and 2, and three speeds.

Figure 5c presents the circuit diagram of the integrated motion‐streak neuron and R–C kernel when applying a 4 V bias voltage, using a 1 MΩ resistor and a 500 pF capacitor to classify the given complex scenarios. Here, the motion‐streak neuron modulates the length of the motion trail according to the length of the optical input, while the R–C kernel detects the interval between two optical input sequences. Longer intervals charge the capacitor more, leading to a higher voltage distribution upon subsequent optical inputs, thereby enabling detection of the interval between consecutive sequences [38].

Figure 5d shows the processed motion‐streak and kernel vectors for two representative complex movement scenarios. When each result is vectorized into a 7500 × 1 vector, the kernel vectors capture distinct features relative to the motion streak vectors. This performance is due to the varying intervals between optical sequences across pixels, resulting in unique vectors when the voltage distribution induced by the final optical input is measured. Based on these characteristics, recognition performance for complex movements was evaluated using both vectors jointly and individually.

Figure 5e presents test accuracy as a function of training epoch for three cases of vectorized inputs. The green curve corresponds to using the motion streak vector alone, yielding a low accuracy of 23.7% because it cannot process input sequences. Also, the decay of relaxation dynamics limits the ability to preserve motion history over the long term. In contrast, using the kernel vector alone yields 54.2% accuracy in recognizing complex movements. It reflects the intervals between optical sequences, but it cannot capture speed‐dependent motion trails. When both vectors are used simultaneously, information about object identity, complex movement, and speed is encoded in a unique combination, resulting in 91.1% accuracy. Figure S33 shows the distributions of the visualized weights before and after training based on these vectors.

Figure 5f shows the predicted output neuron indices for three arbitrary scenarios based on the combined processed vectors, confirming that the activated output neurons correctly infer the ground truth. Figure S34 shows the confusion matrix comparing the 384 true labels with the predicted labels. Lastly, Figure 5g presents the recognition accuracy for the three vectorized input cases as scenarios are added cumulatively. Across all scenario combinations, using both vectors consistently produce higher accuracy than using either vector alone.

## 3 Conclusion

This work demonstrates that a memristive motion‐streak neuron intrinsically encodes spatiotemporal information through the serial integration of an Al/InGaZnO/Al optomemristor and an Ag/HfO_2_/Pt volatile dynamic memristor. This integration enables simultaneous recognition of the object direction, position, and speed within a single neuromorphic system. The implemented hardware comprised a 4 × 4 motion‐streak neuron array for single‐object detection, achieving 96.7% accuracy. The system achieves 96.2% accuracy in identifying the positions, directions, and velocities of multiple moving objects in a simulation of the KTH dataset. Additionally, integration with an R–C kernel enables temporal‐interval processing, achieving 91.1% accuracy and allowing classification of more complex motion sequences within the same hardware architecture. Table 1 compares this work with previously reported memristive motion‐detection sensors. Earlier studies processed only optical inputs and performed single‐object detection on simple datasets [8, 39]. In contrast, this work enables multiple‐object detection on a complex video dataset using an integrated device‐array structure, achieving accuracy comparable to that of the software solution (Figure S35).

**TABLE 1:** Comparison of motion detection across various devices.

	This work	Software	Chen et al. [8].	Song et al. [40].	Sun et al. [39].
Device structure	Al/IGZO/Al + Ag/HfO_2_/Pt	—	MoS_2_‐based phototransistor	Conventional phototransistor (HW5P‐1) + Ag/TiO_x_/Pt	Au/ZnO:N/IGZO/TiN
Device behavior	Optic + dynamic	—	Optic	Optic + dynamic	Optic
Motion dataset	KTH (video, complex)	KTH (video, complex)	Ball movement (video, simple)	NGSIM (video, complex)	Weizmann (frame, simple)
Multiple object detection	yes	—	No	no	no
Accuracy (%)	96.2	98.6	99.2	87.0	97.2

## 4 Experimental Section

### 4.1 Device Fabrication

For the Al/IGZO/Al optomemristor, a SiO_2_ layer was thermally grown on the p‐type (100) Si wafer as a starting substrate. Then, an a‐IGZO layer of 10 nm was deposited at room temperature by RF sputtering using an IGZO compound target under 600 W in Ar gas of 60 standard cubic centimeters per min. The In:Ga:Zn ratio of the target was 1:1:1, which was also the case in the deposited film. The IGZO layer was patterned through photolithography and dry etching. A 50‐nm‐thick Al electrode layer and a 50‐nm‐thick Pt passivation layer were deposited using an electron beam evaporator, followed by the lift‐off process. A line cell structure was designed to maximize the light‐receiving area.

The top electrode of the Ag/HfO_2_/Pt dynamic memristor was connected via the right metal Al electrode of the Al/IGZO/Al optomemristor, and the details of the fabrication process are described elsewhere [41, 42]. A 50‐nm‐thick Pt bottom electrode metal layer was deposited by electron‐beam evaporation and patterned by photolithography and lift‐off. Then, an 8‐nm‐thick HfO_2_ switching layer was deposited through thermal atomic layer deposition at a substrate temperature of 250°C. The precursor for Hf and oxidizer were tetrakis(dimethylamido) hafnium and O_3_, respectively. The HfO_2_ layer was patterned by photolithography and dry etching of the bottom Pt metal electrode of the dynamic memristor and the right metal Al electrode of the optomemristor for pad contact. Finally, a 70 nm thick Ag top electrode layer and a 50 nm thick Pt passivation layer were deposited using a thermal evaporator and an electron‐beam evaporator, respectively, and patterned by photolithography and lift‐off. Figure S12 illustrates the detailed process flow of the integrated motion streak neuron.

### 4.2 Optical Measurements

A custom optical measurement system was constructed to quantify the spatiotemporal response of the 4 × 4 wire‐bonded motion‐streak neuron arrays. Figure 3a represents the schematic of the optical board setup. The optical path was aligned using a 4 × 4 laser array comprising 532 nm green laser diodes with a light intensity of 12.35 mW/cm^2^ (Q‐LINE). Each laser diode was fixed to a laser diode mount (Q‐LINE), and the light was aligned using mirror mounts (Startnow) with 25 mm Si reflective mirrors (FONLAND). The optical modules were assembled on an aluminum optical board (AUTOMATION PARTS, PL23) to ensure stable multilayer beam alignment. The reflected laser beams from the multilayer modules were focused onto the motion‐streak neuron devices (devices under test, DUTs) located at the focal plane.

For synchronized optical illumination and electrical readout, a microcontroller unit (STM32, STMicroelectronics) was connected to each laser channel via bipolar junction transistors (2N2222). The microcontroller unit (MCU) was interfaced with a control computer via a general‐purpose interface bus (GPIB) cable, enabling optical pulse generation in response to a control‐voltage waveform. Through the same GPIB interface, the pulse generator triggered the readout‐voltage window, while the MCU switched the transistor within this window to precisely control the illumination pulse width and to perform automated timing control and data acquisition. Relaxation currents were measured simultaneously using multiple oscilloscope channels connected to the bonded pads of each device, allowing parallel acquisition of spatiotemporal responses. Figure S18 shows the actual hardware configuration used to detect light in different directions.

### 4.3 Electrical Measurements

The DC I–V characteristics were measured using the semiconductor parameter analyzer (4155A, Hewlett‐Packard). An Agilent 8110A pulse generator and oscilloscope (TDS 684C, Tektronix) were used for the pulse measurements. White‐light illumination was provided by a pulse‐width modulator (ZK‐PP2K dual‐mode pulse driver) and a light‐emitting‐diode (LED) lamp module (XML‐T6 5 W LED module).

### 4.4 Movement Classification Methods (Single Object and Multiple Objects Using the KTH Dataset)

For the single‐object case, a neural network with a size of 16 × 24 was trained on 240 experimentally measured responses from the reflective optics assembly, divided into 144 training datasets and 96 test sets. To generate customized datasets from the KTH motion dataset, the original video sequences were first processed with a motion‐detection threshold to extract regions that contain meaningful motion events. The extracted motion sequences were then binarized and resized from the original resolution of 160 × 120 pixels to 100 × 75 pixels. For the multiple‐object classification task, classes were defined as unique combinations of object identity, motion direction, and speed. Each combination was assigned a corresponding digit index and represented as a one‐hot‐encoded output vector. To train on arbitrary scenarios involving multiple objects, a neural network with a structure of 7500 × 48 was trained using 1920 training samples and 480 test samples, corresponding to an 8:2 train‐test split. For more complex scenarios, a neural network with a 15000 × 384 structure was trained on 7680 training samples and 1920 test samples, using an 8:2 train‐test split. The training and test samples were generated by combining preprocessed motion sequences with different object identities, motion directions, and speed conditions, enabling the construction of diverse multi‐object motion scenarios. In both systems, backpropagation was used for the training and testing processes, and the Adam optimizer was applied to minimize the cost function in the Python package PyTorch.

## Author Contributions

**Hyun Wook Kim**: conceptualization, methodology, investigation, validation, visualization, formal analysis, data curation. **Min Chung Jung**: validation. **Néstor Ghenzi**: validation. **Jin Hong Kim**: conceptualization, investigation, methodology, validation, data curation, formal analysis, visualization. **Dong Hoon Shin**: validation. **Sung Keun Shim**: validation. **Joon‐Kyu Han**: validation. **Hyungjun Park**: conceptualization, methodology, data curation, formal analysis, validation, investigation, visualization. **Kyung Seok Woo**: validation. **Cheol Seong Hwang**: conceptualization, methodology, writing – original draft, writing – review and editing, project administration, funding acquisition, supervision.

## Conflicts of Interest

The authors declare no conflicts of interest.

## Supporting information




**Supporting File 1**: adma74793‐sup‐0001‐SuppMat.docx.

**Supporting File 2**: adma74793‐sup‐0002‐VideoS1.mp4.

**Supporting File 3**: adma74793‐sup‐0003‐VideoS2.mp4.

## Data Availability

The data that support the findings of this study are available from the corresponding author upon reasonable request.
