# Supplementary material for: Memristive Motion‐Streak Neuron for Spatiotemporal Multiple Object Detection
Source: Adv Mater. 2026 Aug 22;38(59):e74793. doi: 10.1002/adma.74793 (PMC13689894; doi:10.1002/adma.74793)
Supplement: Supplementary file 1 [file ADMA-38-e74793-s003.docx]

Supplementary Information

Memristive Motion-Streak Neuron for Spatiotemporal Multiple Object Detection

*Hyungjun Park^1^**^†^, Jin Hong Kim^1†^, Hyun Wook Kim^1†^, Néstor Ghenzi^1^, Sung Keun Shim^1^, Joon-Kyu Han^1^, Min Chung Jung^1^, Dong Hoon Shin^1^, Kyung Seok Woo^2,3^, and Cheol Seong Hwang^1*^*

***^1^****Department of Materials Science and Engineering and Inter-University Semiconductor Research Center, Seoul National University, Seoul 08826, Korea*

*^2^Graduate School of Semiconductor Materials and Devices Engineering, Ulsan National Institute of Science and Technology (UNIST), Ulsan 44919, Republic of Korea; Department of Materials Science and Engineering, Ulsan National Institute of Science and Technology (UNIST), Ulsan 44919, Republic of Korea*

***^3^****Department of Materials Science and Engineering, Ulsan National Institute of Science and Technology (UNIST), Ulsan 44919, Republic of Korea*

^*^Correspondence: C.S.H. (cheolsh@snu.ac.kr)

Figure S1. Current-voltage (*I-V*) characteristics of Ag/HfO_2_/Pt dynamic memristor. (a) cumulative probability of set voltages. (b) Set voltages of five devices, each swept over 20 cycles, and (c) their experimental data.

**Figure S2. Relaxation dynamics in the dynamic memristor with varying pulse lengths.** (a) Output current and (b) relaxation behavior of the dynamic memristor with varying pulse lengths under a fixed pulse voltage of 2.8 V and a load resistance of 10 MΩ.

**Figure S3. Relaxation dynamics in the dynamic memristor with varying ambient temperatures.** (a) Relaxation behavior of the dynamic memristor at various ambient temperatures under a fixed pulse voltage condition (2.8 V and 2.5 ms) and a load resistance of 10 MΩ. (b) Derivation of activation energy from relaxation times at different ambient temperatures.

**Figure S4. Relaxation dynamics in the dynamic memristor under varying serial resistances.** Relaxation behavior of the dynamic memristor at different pulse lengths: (a) 1.0 ms, (b) 1.5 ms, (c) 2.0 ms, (d) 2.5 ms, (e) 3.0 ms, (f) 3.5 ms, and (g) 4.0 ms. The pulse amplitude was fixed to 2.8 V.

Figure S5. Relaxation dynamics in the dynamic memristor under varying pulse lengths. Relaxation behavior of the dynamic memristor at different serial resistances: (a) 10 MΩ, (b) 20 MΩ, (c) 30 MΩ, and (d) 50 MΩ. The pulse amplitude was fixed to 2.8 V.

Figure S6. Extracted relaxation times for pulse widths of 2 to 10 ms and pulse heights of 2.8 to 4.0 V. The relaxation time increases monotonically with pulse width and remains clearly distinguishable across the measured range. Error bars represent the standard deviation obtained from ten repeated measurements.

**Figure S7. Band diagrams of AIA optomemristor.** Trap energy levels at light-off and light-on conditions are 0.56 ± 0.02 eV and 0.57 ± 0.02 eV, respectively. The unchanged $q\phi_{T}$ indicates that these traps act as stable centers for capturing photo-excited electrons, hindering recombination of the electron-hole pair and generating increased photocurrent.

 **Figure S8. Temperature dependence of AIA optomemristor.** Ln (*J*/E) vs *E*^0.5^ plot at various temperatures on light-off and light-on conditions. Trap energy levels at light-off and light-on conditions are 0.56 ± 0.02 eV and 0.57 ± 0.02 eV, respectively.

 **Figure S9. Output current of Al/IGZO/Al optomemristor at different light intensities.** The measured data (black dots) and the fitted curve (red dashed line) demonstrate a linear relationship between output current and light intensity.

**Figure S10. Long-term stability of Al/IGZO/Al optomemristor.** (a) Endurance test of light response for 1.5 seconds. Light pulse is applied at fixed intensity and pulse length of 12.35 mW/cm^2^ and 2 ms, respectively, under an applied voltage of 2.8 V. (b) Stability test of light response. Light-on and light-off states are maintained for 100 ms under a fixed voltage of 2.8 V.

**Figure S11. Output current levels measured during repeated light switching cycles for the light-on and light-off states.** The distinct separation of current levels under illumination and in the dark confirms the device's stable and reliable optical switching behavior.

**Supplementary Note 1. Electrical conduction mechanism of the Al/IGZO/Al optomemristor with and without light**

**Figures S7 and S8** present the band diagrams of the Al/IGZO/Al optomemristor and the corresponding conduction mechanisms derived from the fitting results. To further analyze the effect of light, temperature-dependent measurements were conducted under light-off and light-on conditions over the temperature range 303 K to 353 K and the voltage range 2.8 to 3.4 V.

The experimental data follow the log (*J*/*E* vs. *E^0.5^*) relationship, indicating that Poole-Frenkel (P-F) emission is the dominant conduction mechanism in this region^[S1]^. The following equation describes P-F emission:

$J=q\mu N_{c}Eexp\left[ \frac{-q(\phi_{T}-\sqrt{qE/\pi\varepsilon_{r}\varepsilon_{0}})}{kT} \right]$	(S1)

, where $q$ is the electronic charge, $\mu$ is the electronic drift mobility, $N_{c}$ is the density of states in the conduction band, $E$ is the electric field across the dielectric film, $k$ is Boltzmann’s constant, $q\phi_{T}$ ($\Phi_{T}$) is the trap energy level, $\varepsilon_{0}$ is the permittivity in a vacuum, and $\varepsilon_{r}$ is the optical dielectric constant^[S2]^. The extracted trap energy levels were 0.56 ± 0.02 eV and 0.57 ± 0.02 eV under light-off and light-on conditions, respectively. The unchanged $q\phi_{T}$ indicates that these traps act as stable centers for capturing photo-excited electrons, hindering recombination of the electron-hole pair and generating increased photocurrent^[S3], [S4]^.

**Figure S12. Fabrication process flow of the integrated motion streak neuron.**

**Figure S13. *I*-*V* characteristics of Ag/HfO_2_/Pt dynamic memristor in an integrated structure.** (a) *I*-*V* curves of the dynamic memristor and (b) cumulative probability of set voltages. (c) Set voltages of five devices, each swept over 20 cycles, and (d) their experimental data.

Figure S14. AC endurance of the integrated Ag/HfO_2_/Pt dynamic memristor and Al/IGZO/Al optomemristor.

Figure S15. Device-to-device variation of relaxation dynamics in the motion streak neuron with varying pulse lengths. The voltage pulse was set to 4 V during the light-on condition. (a) Output current at varying pulse lengths across 16 pixels. (b) Relaxation time at varying pulse lengths across 16 pixels.

Figure S16. Output current variations in motion streak neurons with varying pulse lengths. The voltage was set to 4 V during the light-on condition.

Figure S17. Relaxation time variation in motion streak neurons with varying pulse lengths. The voltage was set to 4 V during the light-on condition.

Supplementary Note 2. GPIB-based Laser Module Driving and Timing Control Scheme

In this study, a general-purpose interface bus (GPIB)-controlled laser-driving scheme was employed to precisely control the optical stimulation conditions for the memristive motion-streak neuron. A high-power laser module was driven through a bipolar junction transistor (BJT) configured as a current-switching element. In this configuration, a bias voltage was applied to the laser module's anode to provide a stable voltage source suitable for laser illumination, while the collector terminal was connected to the laser module's cathode. The transistor's base terminal was driven by digital output signals from a microcontroller unit (MCU). By modulating the pulse length and timing of the optical stimulation, the transistor was selectively switched between the on and off states, enabling precise control of the laser emission. The laser output was directed toward the device under test (DUT) via a multilayer reflective-optics assembly. By adjusting the optical path and reflection geometry, both the incidence direction and scanning trajectory of the laser could be systematically modulated.

Figure S18. Optical board setup for motion streak neuron hardware.

Optical and electrical pulses were applied synchronously via a GPIB-controlled pulse generator. The reflected laser beams from the multilayer optics assembly were directly applied to the wire-bonded motion-streak neurons (device under test). The oscilloscope captured relaxation responses from multiple DUTs under sequential optical stimulation.

**Figure S19. A light pulse measurement system for observing the relaxation time of motion streak neurons based on the pulse length.** A light pulse is triggered when a voltage pulse is applied to channel 1, and the measured current is read from channel 2 of the oscilloscope.

**Figure S20. Experimental validation of multi-pixel operation under repetitive optical stimulation.**
(a) Experimental measurement setup for the integrated device under repetitive 3 ms optical pulse trains. An external power supply and laser driver were used to generate repetitive optical pulses to the device. (b) Measured current responses showing stable and repeatable operation under continuous optical stimulation, confirming the reliability of the devices for continuous video-event processing.

 **Figure S21. The current responses observed in neighboring pixels due to optical crosstalk.** (a) Measured optical crosstalk induced in adjacent pixels when laser pulses were applied to Pixel 1. (b) Optical crosstalk as a function of distance from pixel 1, where pixels located at the same distance from the illuminated pixel were grouped together and indexed accordingly. The measured current values were fitted with an exponential decay function, and the fitted curve is shown in red. (c) Current responses of neighboring pixels induced by optical crosstalk under a 3 ms optical pulse (1 to 4 ms) applied to the illuminated pixel.

 **Figure S22. Optical crosstalk metrics used for quantitative evaluation.** The crosstalk ratio (CR), total crosstalk ratio (TCR), and signal-to-noise ratio (SNR) were estimated according to the equations listed above. The current change (Δ*I*) is defined as the difference between the measured current response and the dark current (*I*_dark_). Here, $\sigma_{dark}$ denotes the standard deviation of the current measured under dark conditions (without illumination).

**Figure S23. Characterization of timing jitter and spatial alignment in the optical-board setup.** (a) Analysis of the factors contributing to the timing jitter of the pulse generation system. (b) Definition of the spatial alignment ratio based on the normalized distance between the laser spot centroid and the center of the device active layer. (c) Spatial alignment ratio evaluated at each device position to verify the alignment consistency and reproducibility of the optical-board setup.

**Supplementary Note 3. Quantitative relationship between object velocity, optical pulse length, and relaxation behavior**

 In the proposed motion-streak neuron, the object speed is encoded by the optical pulse length applied to each pixel. Since the optical pulse length per pixel is set equal to pixel dwell time, *t*_pulse_ = *t*_dwell_, so that the pulse length is inversely proportional to the object velocity *v* and the spatial interval between pixels defined as *d*:

$t_{pulse}= \frac{d}{v}\propto\frac{1}{v}$	(1)

Based on this definition, pulse lengths of 1, 2, and 3 ms per pixel were chosen to represent fast, medium, and slow speeds, respectively. Also, the relationship between the output current decays and the time constant τ is defined as below:

$I(t)=I_{\mathrm{off}}+(I_{\mathrm{on}}-I_{\mathrm{off}})\text{ }e^{-t/\tau}$	(2)

, where *I*_on_ and *I*_off_ are the currents at light pulse off and after relaxation, respectively. Because a longer pulse forms a thicker silver filament that requires more time to rupture, τ increases linearly with a proportional constant *k*:

$\tau=\tau_{0}+k\cdot t_{pulse}$	(3)

Since the motion streak corresponds to the persistence of the relaxation, its effective length can be expressed as follows :

$t_{streak}=\tau\cdot ln\left[ \frac{I_{on}-I_{off}}{I_{th}-I_{off}} \right]\propto\tau$	(4)

, where *I*_th_ is the current threshold. Therefore, equations (1) to (4) define that a faster object yields a shorter streak, while a slower object yields a longer streak with a stronger residual current at readout.

Figure S24. Measured optical responses of multiple motion streak neurons stimulated by light pulses moving in left, right, and diagonal directions.

Figure S25. Single object movement classification using reflective optics assembly.
The test accuracy saturates at 96.67%, demonstrating the motion-streak neuron's ability to reliably distinguish arbitrary motion velocities and directions.

Figure S26. Evaluation of optical crosstalk between pixels under simultaneous multi-object motion. (a) Measured current responses from non-crossed pixels, showing negligible optical crosstalk when both paths are activated simultaneously. (b) Current responses measured from crossed pixels with different temporal delays between path 1 and path 2, demonstrating the progressive separation of superposed signals as the delay increases.

Figure S27. Flattened output vectors (7500 × 1) derived from motion streak neuron responses for three visual scenarios.
The results are shown for both the frame-based and static relaxation–based cases, where the responses of motion streak neurons are rearranged into a one-dimensional form for comparison.

Figure S28. Experimental current response and corresponding fitted result obtained using the detailed device modeling and simulation methodology. The simulated current response (blue) closely matches the experimentally measured response (red).

**Supplementary Note 4. Detailed device modeling and simulation methodology**

Detailed modeling equations of the device can be described as a first-order ordinary differential equation (ODE) with a state variable $w\in[0, 1]$:

$\frac{dw}{dt}=k\text{ }\text{∙}\text{ }g(V)\text{ }(1-w)\text{ }-\text{ }\frac{w-w_{0}}{\tau}$	(1)

$I=[\text{ }G_{\mathrm{off}}+w\text{ }(G_{\mathrm{on}}-G_{\mathrm{off}})\text{ }]\text{ }V$	(2)

, where $k$ is the control coefficient, $g(V)$ ($=max\left( 0,V-V_{th} \right)$) is the activation function, $\tau$ is the time constant, and $G_{\mathrm{on}}$ and $G_{\mathrm{off}}$ is the high and low conductance extracted from the fabricated device, respectively.

Equation (1) is linear in terms of $w$ under constant bias, $g(V)$ and $w_{ss}$ can be defined as:

$\frac{1}{\tau_{eff}}= k\cdot g(V)+\frac{1}{\tau}$ , $w_{ss}= \tau_{eff}\left[ k\cdot g(V)+\frac{w_{0}}{\tau} \right]$	(3)

Therefore, the response approaches the steady-state value exponentially.

$w(t)=w_{\mathrm{ss}}+(w_{\mathrm{initial}}-w_{\mathrm{ss}})\text{ }e^{-t/\tau_{\mathrm{eff}}}$	(4)

Each stage then follows by inserting the appropriate $g(V)$ and mapping $w$ to current through equation (2).

Without stimulus, $g(V)\to0$, resulting in $\tau_{\mathrm{eff}}=\tau$ and $w_{\mathrm{ss}}=w_{0}$. For the on-state $w_{\mathrm{on}}$, equation (2) therefore gives the relaxation:

$I(t)=I_{\mathrm{off}}+(I_{\mathrm{on}}-I_{\mathrm{off}})\text{ }e^{-t/\tau}$	(5)

Under stimulus, $g(V)=g(V_{\mathrm{pulse}})>0$, the efficient constant can be derived as $\tau_{rise}=\frac{1}{k\cdot g\left( V_{pulse} \right)+\frac{1}{\tau}}$ and $w_{\mathrm{ss}}=w_{\mathrm{on}}$. For $w_{\mathrm{initial}}\cong0$, equation (2) therefore gives the current response:

$\text{ }I(t)=I_{\mathrm{off}}+(I_{\mathrm{on}}-I_{\mathrm{off}})(1-e^{-t/\tau_{\mathrm{rise}}})$	(6)

Figure S29. Visualization of the learned weight distributions across output neuron indices after training for (a) the frame-based and (b) static relaxation–based processing.
Each heatmap shows the magnitude of the weight assigned to each output neuron for each vector.

Figure S30. Flattened output vectors (7500 × 1) derived from three arbitrary visual scenarios, where the output neuron index encodes object identity, motion direction, and speed. The resulting activation patterns represent the classification outcome for the frame-based and static relaxation–based processing schemes.

Figure S31. Confusion matrix illustrates the classification results for multiple moving-object scenarios (two object identities (O1, O2), eight motion directions, and three distinct speed levels). The diagonal structure confirms high classification fidelity, demonstrating that the proposed system effectively resolves object identity, directionality, and dynamic motion speeds across arbitrary visual conditions.

Figure S32. Variation in test accuracy over ten trials with respect to the coefficient of variation for 48 labels (2 object identities, 8 directions, 3 movement speeds). Despite the increasing standard deviation relative to the average current value, test accuracy is mostly unaffected. This suggests that the motion streak neuron is resilient to variations in current.

Figure S33. Visualization of the learned weight distributions across output neuron indices after training for arbitrary complex visual scenarios.
Each heatmap shows the magnitude of the weights assigned to individual output neurons for motion streak and RC kernel vectors.

Figure S34. Confusion matrix illustrates the classification results for complex visual scenarios (two object identities (O1, O2), eight motion directions for trajectories 1 and 2, and three distinct speed levels). The diagonal structure demonstrates that the integrated motion streak neuron and RC kernel effectively resolve object identity, complex trajectory, and dynamic motion speeds across arbitrary visual conditions.

Figure S35. Schematic representation of the transition from simulating biological mechanisms using a purely CMOS approach to emulating them with dynamic memristor and optomemristor.

Supplementary Table 1. Comparison of representative device characteristics for motion detection.

|  | **This work** | **Chen et al.**^[S5]^ | **Song et al.**^[S6]^ | **Sun et al.**^[S7]^ | **Han et al.**^[S8]^ |
| --- | --- | --- | --- | --- | --- |
| Device  structure | Al/IGZO/Al +  Ag/HfO_2_/Pt | MoS_2_-based  phototransistor | Conventional phototransistor (HW5P-1) + Ag/TiO_x_/Pt/Ti/NbOx/Pt | Au/ZnO:N/IGZO/TiN | Au/ZnO-UCNPs/ FTO |
| Rising / falling time | 100 μs / 60 μs | - | 100 μs / - | 50 ms / 25 ms | > 20 s |
| Endurance | > 2.0*10^6^ | - | > 10^6^ | - | > 10^4^ |
| Demonstration level | Array-level | Array-level | Device-level with peripheral circuitry | Device-level | Device-level |
| Sampling frequency | 71.5 Hz | 10 Hz | 5000 Hz | 20 Hz | 0.05 Hz |

**References**

[S1]	H.-W. Yeon, S.-M. Lim, J.-K. Jung, H. Yoo, Y.-J. Lee, H.-Y. Kang, Y.-J. Park, M.	Kim, Y.-C. Joo, *NPG Asia Mater.* **2016**, *8*, e250.

[S2]	F.-C. Chiu, C.-M. Lai, *J. Phys. D: Appl. Phys.* **2010**, *43*, 075104.

[S3]	H. Wei, X. Ji, J. Cao, W. He, H. Liu, Z. Pan, X. Song, Q. Sun, J. Li, C. Wu, *ACS	Nano* **2024**, *18*, 26643.

[S4]	M. D. Ward, A. J. Bard, *J. Phys. Chem.* **1982**, *86*, 3599.

[S5]	J. Chen, Z. Zhou, B. J. Kim, Y. Zhou, Z. Wang, T. Wan, J. Yan, J. Kang, J.-H. Ahn, Y. Chai, *Nat. Nanotechnol.* **2023**, *18*, 882.

[S6]	H. Song, M. G. Lee, G. Kim, D. H. Kim, G. Kim, W. Park, H. Rhee, J. H. In, K. M. Kim, *Adv. Mater.* **2024**, *36*, 2309708.

[S7]	Y. Sun, Q. Li, X. Zhu, C. Liao, Y. Wang, Z. Li, S. Liu, H. Xu, W. Wang, *Adv. Intell. Syst.* **2023**, *5*, 2200196.

[S8]	J. Han, H. Mao, Y. Lin, K. Peng, L. Meng, Y. Ding, X. Shan, Y. Tao, J. Shi, Z. Wang, H. Zhu, X. Zhao, H. Xu, Y. Liu, *Adv. Mater.* **2025**, 37, e10185.
